# The association between nurse staffing and hospital outcomes in injured patients

**DOI:** 10.1186/1472-6963-12-247

**Published:** 2012-08-09

**Authors:** Laurent G Glance, Andrew W Dick, Turner M Osler, Dana B Mukamel, Yue Li, Patricia W Stone

**Affiliations:** 1 Departments of Anesthesiology, and Community and Preventive Medicine, University of Rochester School of Medicine, Rochester, NY, USA; 2 RAND, RAND Health, Boston, MA, USA; 3 Department of Surgery, University of Vermont Medical College, Burlington, VT, USA; 4 Department of Medicine, Center for Health Policy Research, University of California, Irvine, CA, USA; 5 University of Iowa, Iowa, IA, USA; 6 Center for Health Policy, Columbia University School of Nursing, New York, NY, USA

## Abstract

**Background:**

The enormous fiscal pressures facing trauma centers may lead trauma centers to reduce nurse staffing and to make increased use of less expensive and less skilled personnel. The impact of nurse staffing and skill mix on trauma outcomes has not been previously reported. The goal of this study was to examine whether nurse staffing levels and nursing skill mix are associated with trauma patient outcomes.

**Methods:**

We used data from the Healthcare Cost and Utilization Project Nationwide Inpatient Sample to perform a cross-sectional study of 70,142 patients admitted to 77 Level I and Level II centers. Logistic regression models were used to examine the association between nurse staffing measures and (1) mortality, (2) healthcare associated infections (HAI), and (3) failure-to-rescue. We controlled for patient risk factors (age, gender, injury severity, mechanism of injury, comorbidities) and hospital structural characteristics (trauma center status - Level I versus Level II, hospital size, ownership, teaching status, technology level, and geographic region).

**Results:**

A 1% increase in the ratio of licensed practical nurse (LPN) to total nursing time was associated with a 4% increase in the odds of mortality (adj OR 1.04; 95% CI: 1.02-1.06; p = 0.001) and a 6% increase in the odds of sepsis (adj OR 1.06: 1.03-1.10; p < 0.001). Hospitals in the highest quartile of LPN staffing had 3 excess deaths (95% CI: 1.2, 5.1) and 5 more episodes of sepsis (95% CI: 2.3, 7.6) per 1000 patients compared to hospitals in the lower quartile of LPN staffing.

**Conclusions:**

Higher hospital LPN staffing levels are independently associated with slightly higher rates of mortality and sepsis in trauma patients admitted to Level I or Level II trauma centers.

## Background

Efforts to improve quality of care for injured patients have included the regionalization of trauma care [1], establishment of benchmarking standards [2], and the creation of regional and national trauma registries [3]. Evidence-based guidelines have been created to standardize treatment and apply best practices to the care of injured patients [4]. Despite these broad-based efforts to improve the quality of trauma care, there remains substantial variation in mortality and complications across hospitals and trauma centers treating injured patients [5,6]. In response, the American College of Surgeons (ACS) has created the Trauma Quality Improvement Program (TQIP) [7] and has closely collaborated on a Agency for Healthcare Research and Quality funded project, the Survival Measurement and Reporting Trial for Trauma (SMARTT) [8], to create hospital performance measures and use hospitals as "learning laboratories" [9] to identify best practices for improving trauma care.

Some of the variability in outcomes may be attributable to differences in physician and nurse staffing across hospitals. According to the American College of Surgeons (ACS) Committee on Trauma (COT), "Optimal resources [at a trauma center include] the immediate availability of board-certified emergency physicians, general surgeons, anesthesiologists, neurosurgeons, and orthopedic surgeons [10]." Over the past decade, physician workforce issues in trauma have attracted increasing attention because the undersupply of trauma surgeons has led to a critical problem in patient access to emergency care [11]. Although nurses are at the front-line of patient care, the ACS COT Resources for Optimal Care of the injured Patient provides very little guidance on nurse staffing and skill mix. According to the Institute of Medicine, "how well we are cared for by nurses affects our health, and sometimes can be a matter of life or death [12]." Many studies have documented that higher levels of nurse staffing are associated with better outcomes and fewer complications for medical and surgical patients [13-17]. However, to our knowledge, the impact of nurse staffing on trauma outcomes has not been previously investigated. The enormous fiscal pressures facing trauma centers [18] may lead trauma centers to reduce nurse staffing and to make increased use of less expensive and less skilled personnel. Our goal in this study is to examine the association between nurse staffing and mortality, healthcare associated infections, and failure-to-rescue [19] using a nationally representative sample of Level I and Level II trauma centers. We hypothesized that lower levels of nurse staffing and increased use of nurses with less training are associated with worse outcomes in injured patients. Findings from this study may help inform decisions about nurse staffing levels in hospitals caring for injured patients.

## Methods

### Data source

We used data from the 2006 HCUP Nationwide Inpatient Sample (NIS) to perform this study. The NIS, which contains data from a 20% stratified sample of U.S. hospitals, is the largest all-payer hospital inpatient database in the United States. This database includes information on patient demographics, admission source, ICD-9-CM diagnostic and injury codes, AHRQ comorbidity measures [20], in-hospital mortality, hospital characteristics, and hospital identifiers. The NIS data were linked to the 2007 American Hospital Association Annual Survey Database to obtain trauma center designation and staffing information. The University of Rochester School of Medicine Institutional Review Board approved this study after expedited review (Rochester, NY).

### Study population

The study sample consisted of patients admitted with a principal ICD-9-CM diagnosis of trauma (800–959.9), after excluding patients with burns (940–949); unspecified injuries (959–959.9); patients with the following isolated injuries: late effects of injury (905–909.9), superficial injuries (910–924.9), or foreign bodies (930–939.0). We excluded observations with missing demographic information on age, gender, or outcome (1,162); missing Ecodes (External cause-of-injury coding) (8,847); patients with non-traumatic mechanisms (4,504); patients who were transferred to other hospitals (1,377). Patients admitted to hospitals that were affiliated to nursing homes were excluded because the American Hospital Association (AHA) Survey data does not differentiate between hospital and nursing home nurse staffing for hospitals with associated nursing homes (15,971). Observations from hospitals missing information on nurse staffing (6,653) or on the CMS Case-Mix Index (2,176) were also excluded. The final study cohort consisted of 70,142 patients in 77 Level I and Level II hospitals.

### Staffing measures

Staffing measures for RNs, licensed practical nurses (LPN), and nurses' aides (NA) were separately estimated using hours per patient day (HPPD) [13,21] with data from the American Hospital Association (AHA) Survey database. In theory, RN HPPD could be calculated by multiplying the number of hospital RN FTEs by 2,040 hours per year and then dividing by inpatient days. However, this would over-estimate the amount of time spent by nurses caring for inpatients since the AHA survey only reports total nurse staffing, and does not distinguish between nurses working in the inpatient versus outpatient setting. But, the AHA survey also reports "adjusted patient days" which is the sum of inpatient days and an estimate of outpatient days (based on the ratio of outpatient to inpatient revenues) [22]. Thus, in order to avoid over-estimating nursing hours spent caring for inpatients, registered nurse hours per patient day were estimated by multiplying RN FTEs by 2,040 hours per year and then dividing by adjusted patient days (as opposed to dividing by inpatient days), using a standard approach described by Kovner and colleagues [22]. To account for cross-sectional variation in patient acuity, we further adjusted the estimate for RN HPPD by dividing HPPD by the hospital CMS case-mix index as described by Needleman and colleagues [13,22]. The case mix index is a relative measure of patient resource consumption (intensity of care) based on diagnosis related group (DRG) relative weights [23]. Licensed nurse HPPD and nurses' aide Hpdd were estimated in a similar fashion. All staffing measures reflect average staffing across all hospital departments and do not represent staffing on individual units [13,14,22,24].

### Statistical analysis

We estimated separate logistic regression models to examine the association between the three nurse staffing measures (RN HPPD, LPN HPPD, and nurses' aides HPPD) and (1) mortality, (2) healthcare associated infections (HAI), and (3) failure-to-rescue. Failure-to-rescue was defined as death in a patient with a healthcare associated infection. Patients with sepsis, pneumonia, *Staphylococcus infections*, or *Clostridium difficile* associated disease (CDAD) were classified as having an HAI. We used previously published criteria for identifying HAIs using ICD-9-CM codes (Table 1) [25-29]. Patients with length-of-stay less than 3 days were excluded from analyses based on HAIs. Cases identified using these algorithms were assumed to represent HAIs since it is not likely that patients admitted with traumatic injuries would have pre-existing infections.

**Table 1:** Criteria for identifying health-care associated infections

**Infection Type**	**ICD-9-CM Discharge Diagnosis Codes**
Sepsis	038-038.9, 112.5, 112.81, 785.52, 995.91, 995.92
Pneumonia	482.0-482.2, 482.4-482.9
Staphylococcus	730.0-730.09, 711–711.09, 038.11, 041.11, 482.41, V09.0, V09.8, 008.41, 038.1, 790.7, 996.62, 421.0, 996.61, 998.3, 998.5
Clostridium difficile	008.45

We controlled for patient demographics (age and gender), injury severity, mechanism of injury, and comorbidities. Injury severity was coded using empirically-derived estimates of injury severity based on the previously validated Trauma Mortality Prediction Model (TMPM) [30,31]. The AHRQ comorbidity algorithm was used to code patient comorbidities [20]. Fractional polynomial analysis was used to obtain the optimal specification for age [32].

We also controlled for hospital characteristics: teaching status, trauma center status (Level I vs. Level II), hospital ownership, hospital bed size, technology, and geographic region. We classified hospitals with resident-to-bed ratios less than 1:4 as minor teaching hospitals and those with ratios greater than 1:4 as major training hospitals [14]. Hospitals with no residents were considered to be nonteaching hospitals. Hospitals were categorized as small (≤ 250 beds), medium (251–500 beds), and large (> 500 beds). Hospitals that performed either heart or lung transplants were defined as high-technology hospitals.

Direct standardization was used to illustrate the impact of changing LPN staffing on outcomes using our fitted models. We modified our baseline models (described above) to estimate the probability of mortality and healthcare associated infections under two alternative scenarios: (1) bottom quartile of LPN HPPD; and (2) top quartile of LPN HPPD. Ninety-five percent confidence intervals were constructed using bootstrapping [33].

Finally, we modified the baseline models to explore an alternative specification of nurse staffing in order to investigate a possible substitution effect of LPNs for RNs. We defined the staffing ratio of LPN staffing to total nurse staffing (LPN HPPD plus RN HPPD) as:

(1) $$\text{staffing ratio}=\frac{LPN\;hppd}{LPN\;hppd+RN\;hppd}\;\times \;100$$

These model included staffing ratio and total nurse staffing as measures of nurse staffing.

All statistical analyses were performed using STATA SE/MP Version 11.0 (STATA Corp., College Station, TX). Robust variance estimators were used because observations for patients treated at the same trauma center may be correlated [34]. The performance of the logistic regression models were assessed using measures of discrimination (C statistic) and calibration (the Hosmer-Lemeshow statistic).

## Results

### Patient population

Table 2 describes patient demographics. The median age for patients in our study population was 47, with an inter-quartile range between 26 and 72. The majority of patients were male (59.8%). The most common injury mechanisms was blunt trauma (47.1%), followed by motor vehicle accident (18.3%), and low-falls (17.6%). Our sample had an in-hospital mortality rate of 3.1% and an incidence of healthcare associated infections of 2.8%.

**Table 2:** Patient demographics

Number of patients	70,142
Age, median and inter-quartile range	47 (26,72)
Female, No. (%)	28,225 (40.2)
Injury mechanism, No. (%)	
Blunt trauma	33,066 (47.1)
Motor Vehicle Accident	12,824 (18.3)
Gunshot	2,587 (3.69)
Stab	3,684 (5.25)
Pedestrian trauma	5,673 (8.09)
Low-fall	12,308 (17.6)
Outcomes, No. (%)	
In-hospital mortality	2,190 (3.12)
Healthcare Associated Infections, total	1,951 (2.78)
Sepsis	776 (1.11)
Pneumonia	796 (1.13)
Staphylococcus infections	1,044 (1.49)
Clostridium difficile Infection	249 (0.35)

### Hospital characteristics

Hospital characteristics are displayed in Table 3. About half of the hospitals were Level I trauma centers (55%) versus level II trauma centers. About half of the hospitals were teaching hospitals (47%), the majority were not-for-profit (75%), and most did not have either a heart or lung transplant program (83%). Registered nurses provided a median of 6.3 hours of nursing care per day (HPPD), licensed practical nurses 0.20 HPPD, and nurses' aides 1.4 HPPD (Table 4).

**Table 3:** Hospital demographics

		**Hospitals**	**Patients**
Trauma Center Accreditation, No. (%)	Level I	42 (55)	47,350 (67.5)
	Level II	35 (45)	22,792 (32.5)
Hospital Size, No. (%)	Small (<250 beds)	24 (31)	6,343 (9.0)
	Medium (250–500 beds)	34 (44)	33,019 (47.1)
	Large (>500 beds)	19 (25)	30,780 (43.9)
Teaching Status, No. (%)	Major Teaching	23 (30)	33,934 (48.4)
	Minor Teaching	13 (17)	12,996 (18.5)
	Non-Teaching	41 (53)	23,212 (33.1)
Hospital Ownership, No. (%)	Not-for-profit	58 (75)	48,885 (69.7)
	For profit	5 (6.5)	4,312 (6.2)
	Governmental non-federal	14 (18)	16,945 (24.2)
High Technology, No. (%)		13 (17)	19,582 (27.9)
Geographic Region, No. (%) R	Northeast	22 (29)	20,755 (29.6)
	South	19 (25)	17,129 (24.4)
	Mid-West	15 (19)	10,948 (15.6)
	West	21 (27)	21,310 (30.4)

**Table 4:** Nurse Staffing Hours

Hours of nursing care per patient-day	
Registered-nurse, median hours (inter-quartile range)	6.3 (5.3, 7.1)
Licensed-practical nurse, median hours (inter-quartile range)	0.20 (0.09, 0.33)
Nurses' Aide, median hours (inter-quartile range)	1.4 (1.1, 2.3)
Total, median hours (inter-quartile range)	7.9 (6.9, 9.2)
Total hours of Licensed Nursing care per day, median hours (IQR) range)	6.6 (5.7, 7.4)
Proportion of Total Licensed Nursing Time (%)	
Registered-nurse, median % (inter-quartile range)	96.9 (95.0, 98.4)
Licensed-practical nurse, median % (inter-quartile range)	3.1 (1.6, 5.0)

IQR - inter-quartile range.

### Association between nurse staffing and adverse outcomes

Tables 5 and 6 displays the results of our analyses of the effects of RN, LPN, and nurses' aide staffing on mortality, healthcare associated infections, and failure-to-rescue. Adjusting for patient demographics, injury severity, mechanism of injury, comorbidities, and hospital characteristics (teaching status, trauma center status, hospital ownership, hospital bed size, technology, geographic region), we found that a one-quarter hour increase in LPN hours per patient day was associated with a 15% increase in the odds of death (adj OR 1.15; 95% CI: 1.05, 1.25; p = 0.002) and a 27% increase in the odds of sepsis (adj OR 1.27; 95% CI: 1.11, 1.45; p < 0.001). However, this effect size should be interpreted in the context that LPNs provided, on average, only 0.20 HPPD.

**Table 5:** Odds ratios estimating the association between nurse staffing on patient mortality, healthcare associated infections, and failure to rescue

	**Unadjusted OR**^**c**^**(95% CI)**	**P Value**	**Adjusted**^**d**^**(95% CI)**	**P Value**
Mortality				
RN staffing ^a e^	0.99 (0.98, 1.00)	0.12	0.99 (0.98, 1.00)	0.12
LPN staffing ^a e^	0.91 (0.81, 1.02)	0.12	1.15 (1.05, 1.25)	0.002
Nurses' Aide staffing ^a e^	0.99 (0.97, 1.02)	0.60	0.99 (0.96, 1.02)	0.42
% Ratio of LPN to Total Nursing^b^	0.98 (0.95, 1.01)	0.21	1.04 (1.02, 1.06)	0.001
Total Nursing Time^b^	0.95 (0.90, 1.00)	0.034	0.99 (0.95, 1.03)	0.49
Hospital-Associated Infection				
RN staffing	1.01 (0.98, 1.03)	0.55	1.02 (0.99, 1.04)	0.14
LPN staffing	0.96 (0.83, 1.10)	0.54	1.15 (0.998, 1.33)	0.053
Nurses' Aide staffing	0.96 (0.93, 0.99)	0.021	0.97 (0.93, 1.01)	0.091
% Ratio of LPN to Total Nursing	0.99 (0.95,1.02)	0.45	1.03 (1.00, 1.07)	0.08
Total Nursing Time	1.02 (0.93, 1.12)	0.66	1.08 (0.99, 1.18)	0.07
Failure-to-Rescue				
RN staffing	0.99 (0.96, 1.01)	0.35	1.01 (0.99, 1.03)	0.45
LPN staffing	0.90 (0.74, 1.10)	0.30	1.19 (0.94, 1.52)	0.16
Nurses' Aide staffing	1.02 (0.96, 1.08)	0.57	0.99 (0.93, 1.06)	0.74
% Ratio of LPN to Total Nursing	0.98 (0.94,1.03)	0.54	1.04 (0.98, 1.10)	0.18
Total Nursing Time^b^	0.94 (0.85, 1.04)	0.26	1.05 (0.95, 1.17)	0.32

^a^ results based on model which included RN staffing and LPN staffing as measures of nurse staffing.

^b^ results based on model which included staffing ratio and total nurse staffing as measures of nurse staffing.

^c^ In the unadjusted analyses, only the nurse staffing measures were included in the regression models.

^d^ adjusted for patient demographics, injury severity, mechanism of injury, comorbidities, and hospital characteristics (teaching status, trauma center status, hospital ownership, hospital bed size, technology, geographic region).

^e^ staffing is in 15-minute increments per patient day (0.25 HPPD).

**Table 6:** Odds ratios estimating the association between nurse staffing and individual hospital-associated infections

	**Unadjusted**^**c**^**(95% CI)**	**P Value**	**Adjusted**^**d**^**(95% CI)**	**P Value**
Sepsis				
RN staffing^a^	1.00 (0.97, 1.03)	0.97	1.01 (0.99, 1.03)	0.37
LPN staffing^a e^	0.98 (0.87, 1.10)	0.77	1.27 (1.11, 1.45)	<0.001
Nurses' Aide staffing^a^	0.97 (0.93, 1.01)	0.113	1.00 (0.96, 1.03)	0.81
% Ratio of LPN to Total Nursing^b^	1.00 (0.97, 1.03)	0.99	1.06 (1.03, 1.10)	<0.001
Total Nursing Time^b^	1.00 (0.90, 1.11)	0.96	1.07 (1.00, 1.14)	0.07
Pneumonia				
RN staffing	1.01 (0.97, 1.05)	0.74	1.03 (0.99, 1.07)	0.17
LPN staffing	0.78 (0.61, 0.99)	0.043	1.00 (0.78, 1.30)	0.98
Nurses' Aide staffing	0.95 (0.89, 1.01)	0.096	0.92 (0.87, 0.98)	0.006
% Ratio of LPN to Total Nursing	0.94 (0.88, 0.99)	0.02	0.99 (0.93, 1.06)	0.86
Total Nursing Time	1.00 (0.84, 1.18)	0.97	1.10 (0.95, 1.30)	0.18
Staphylococcus infections				
RN staffing	1.01 (0.99, 1.04)	0.21	1.02 (1.00, 1.05)	0.056
LPN staffing	1.02 (0.86, 1.21)	0.81	1.15 (0.98, 1.36)	0.083
Nurses' Aide staffing	0.96 (0.93, 1.00)	0.064	0.98 (0.93, 1.02)	0.32
% Ratio of LPN to Total Nursing	0.97 (0.95, 1.04)	0.91	1.03 (0.99, 1.07)	0.19
Total Nursing Time	1.06 (0.96, 1.17)	0.25	1.10 (1.00, 1.21)	0.04
Clostridium difficile infections				
RN staffing	0.97 (0.94, 1.01)	0.16	0.99 (0.96, 1.02)	0.54
LPN staffing	0.73 (0.56, 0.94)	0.014	0.83 (0.65, 1.07)	0.15
Nurses' Aide staffing	1.01 (0.95, 1.07)	0.76	1.00 (0.95, 1.06)	0.98
% Ratio of LPN to Total Nursing	0.94 (0.87, 1.02)	0.12	0.96 (0.90, 1.02)	0.19
Total Nursing Time	0.87 (0.76, 1.01)	0.07	0.94 (0.82, 1.08)	0.19

^a^ results based on model which included RN staffing and LPN staffing as nurse staffing measures.

^b^ results based on model which included staffing ratio and total nurse staffing as measures of nurse staffing.

^c^ In the unadjusted analyses, only the nurse staffing measures were included in the regression models.

^d^ adjusted for patient demographics, injury severity, mechanism of injury, comorbidities, and hospital characteristics (teaching status, trauma center status, hospital ownership, hospital bed size, technology, geographic region).

^e^ staffing is in 15-minute increments per patient day (0.25 HPPD).

There was no significant association between RN staffing and overall outcomes. However, a 1% increase in the ratio of LPN to total nursing time (RN plus LPN HPPD) was associated with a 4% increase in the odds of mortality (adj OR 1.04; 95% CI: 1.02-1.06; p = 0.001) and a 6% increase in the odds of sepsis (adj OR 1.06: 1.03-1.10; p < 0.001). These findings suggest that the increased risk of mortality associated with higher levels of LPN staffing are caused by the substitution of LPNs for RNs (Tables 5 and 6). We also found that a one-quarter hour increase in nurses' aide hour per patient day was associated with a 8% decrease in the odds of pneumonia (adj OR 0.92; 95% CI: 0.87, 0.98; p = 0.006) (Table 6).

The mortality models exhibited excellent discrimination (C statistic = 0.93) and acceptable calibration (HL statistic 63), given the large size of the data set and the HL statistic's well known sensitivity to sample size [35]. The HAI and FTR models exhibited good to very good discrimination (C statistic ranging from 0.75 to 0.87), and acceptable calibration (HL statistic ranging from 7.5 to 37).

Direct standardization was used to predict the number of excess deaths and sepsis associated with different levels of LPN staffing. We found that hospitals in the highest quartile of LPN staffing had 3 excess deaths (95% CI: 1.2, 5.1) per 1000 patients compared to hospitals in the lower quartile of LPN staffing. Similarly, increased LPN staffing was associated with 5 more episodes of sepsis (95% CI: 2.3, 7.6) per 1000 patients.

## Discussion

In this study, based on a large nationally representative sample of Level I and Level II trauma centers, higher LPN staffing levels were independently associated with increased mortality and higher rates of sepsis. Our results indicate that trauma centers with the lowest LPN-to-patient staffing ratios (lower quartile of LPN staffing) would have 3 fewer deaths and 5 fewer episodes of sepsis per 1000 trauma admissions. We found that higher proportion of nursing care provided by LPNs is associated with increased rates of mortality and sepsis, suggesting that substitution of LPNs for RNs may be the mechanism leading to worse outcomes in hospitals with higher levels of LPN staffing. To our knowledge, this is the first study to report on the association between nurse staffing and outcomes in trauma.

Prior studies have shown that nursing skill mix is associated with patient outcomes. Needleman and colleagues reported that higher proportions of nursing care provided by registered nurses (relative to LPNs) is associated with fewer complications and lower failure-to-rescue rates in medical patients [13]. Other investigators have reported that higher levels of LPN staffing are associated with higher mortality in patients with acute myocardial infarctions [24]. Aiken and colleagues found that higher proportions of nurses holding baccalaureate degrees is associated with decreased mortality and likelihood of failure-to-rescue [36]. In its recently released report on *The Future of Nursing*[37], the Institute of Medicine highlights the fact that nurses operate within an increasingly more complex healthcare environment in which nurses interface with complex technologies and collaborate with a large group of highly educated health professionals. Empiric evidence that a more highly educated nursing workforce is associated with better patient outcomes provides support for one the key pillars of the Institute of Medicine report: "nurses should achieve high levels of education and training through an improved education system" in which "a greater number of nurses [will] enter the workforce with a baccalaureate degree or progress to this degree early in their career [37]."

Despite finding that higher LPN staffing is associated with worse outcomes, we did not find that patients in hospitals with higher nurses' aide staffing experienced higher mortality or a greater number of healthcare associated infections. In fact, we found that higher nurses' aide staffing was associated overall with fewer pneumonias. These findings may seem surprising in light of the association between higher educational levels for licensed nurses (e.g. RNs holding bachelor's degree versus RN without a bachelor's degree, RN versus LPN) and better patient outcomes. One possible explanation for these findings is that there is a sufficiently high degree of overlap in the skill mix and scope of practice for LPNs and RNs that less-skilled LPNs are sometimes substituted for RNs, whereas nurses' aides have a very different set of clinical responsibilities compared to licensed nurses (RNs and LPNs), and are therefore not likely to be substituted for RNs. Alternatively, increased use of LPN staffing could be a proxy for a poor nurse work environment, which is associated with increased mortality [38].

This study has several important limitations. First, nurse staffing levels reported in the AHA database represent average staffing across individual hospitals and are not specific to individual patient populations within hospitals (e.g. trauma patients). Aiken and colleagues have argued that "staffing [can be] measured across entire hospitals because there is no evidence that specialty-specific staffing offers advantages in the study of patient outcome … [14]" Furthermore, the use of a global nurse staffing measure, as opposed to specialty-specific nurse measure, does reflect "the fact that patients often receive nursing care in multiple specialty areas of a hospital [14]." In addition, nurse staffing measures do not reflect actual time spent at the bedside and do not discriminate between patient-related activities and administrative functions [22]. In practice, nurses at two hospitals reporting identical results for nurse hours per patient day may actually spend different amounts of time at the patient bedside.

Second, we cannot rule out the possibility that residual confounding, due to unmeasured severity-of-illness, accounted for the observed association between nurse staffing and outcomes. Our trauma mortality prediction model is based on administrative data and therefore does not include important information on patient physiology such Glasgow coma scale and vital signs on presentation. However, our trauma mortality prediction model (TMPM-ICD9), developed as part of an AHRQ-funded program to evaluate the impact of non-public report cards for trauma, has been previously validated and found to have excellent statistical performance [30]. Third, the association between nurse staffing and outcomes may be spurious due to unmeasured variation in hospital policies. For example, hospitals might respond to financial pressures in a variety of ways that could affect quality, including adjustments in the nurse staffing mix. Nurse staffing, therefore, might act as a proxy for other unobserved behavior that affects mortality and infection rates. This concern is limited by the fact that we control for hospital governance, teaching status, and size. In addition, work by Blegen and colleagues has shown that LPN staffing increases in areas where RN supply is lower [21], indicating that staffing is driven at least in part by considerations that are external to the hospitals. Fourth, administrative data do not always distinguish between pre-existing conditions and complications [39]. Although it is possible that some of the infections identified as healthcare associated infections were community-acquired, it is very likely that most infections in trauma patients are hospital acquired.

This study may have potential policy implications. The extent to which patient acuity and nursing skill mix are appropriately matched may warrant additional study. The work by Blegen and colleagues, which demonstrates the relationship between nurse supply and LPN staffing, highlights the concern about potential future nurse workforce shortages [21]. Although the nursing workforce shortage [40] has ended, the recent expansion of the RN workforce may represent at temporary “bubble” due to the loss 7.5 million jobs in the broader economy [41]. If this recent expansion is a temporary bubble and eventually leads to a post-recession shortage [41], then hospitals may respond to possible future shortages in nursing workforce by substituting LPNs for RNs [40]. The evidence suggests that dealing with nursing shortages by increasing the proportion of less skilled nurses may have unintended consequences.

## Conclusions

This study is the first study to demonstrate that nurse staffing skill mix may be associated with patient outcomes in trauma patients. It provides additional empirical evidence to support the association between nurse staffing and patient outcomes across all patient populations. In light of the many challenges facing trauma centers and the trauma community [18], exploring potential solutions to future nursing shortages may be especially important. As recommended by the IOM, better data collection and information infrastructure is necessary [37], and such data may help guide efforts to reengineer nursing processes to make health care more efficient and safer for all patients. Projected deficits in the nursing workforce, and the critical role of nurses in ensuring patient safety, make it imperative that we gain a better understanding of how to best optimize the nursing workforce - their role, training, and work environment - in order to address the needs of an increasingly more complex patient population.

## Competing interest

The authors have no competing interests.

## Authors’ contributions

LGG, AWD, and PWS developed the conceptual model and study design. LGG performed the compilation and synthesis of the data. LGG and AWD carried out the statistical analysis. LGG supervised the research project. All authors participated in interpretation of the results and writing of the report, and approved the final version of the manuscript.

## Pre-publication history

The pre-publication history for this paper can be accessed here:

http://www.biomedcentral.com/1472-6963/12/247/prepub
